# Commentary: A Novel Approach to Breast Ptosis in Reduction Mammoplasty: Outcomes of the Paisley Pattern Incision

**DOI:** 10.1177/22925503261485233

**Published:** 2026-09-10

**Authors:** Martin R. LeBlanc

**Affiliations:** 1 Division of Plastic Surgery, 3688 Dalhousie University, Halifax, Nova Scotia, Canada

The evolution of breast reduction over the past several decades has seen the adoption of the Wise-pattern breast reduction with inferior pedicle, followed by the vertical reduction with superior medial pedicle. The latter technique was developed to try and reduce bottoming out made worse by use of an inferior pedicle and reducing scarring with the vertical-only incision. Use of the superior pedicle may help with bottoming out. However, I and others have found it hard to eliminate the deformity or redundant tissue at the bottom of the vertical incision. Anecdotally, the use of the superior medial pedicle and Wise pattern has significantly improved my results in part by eliminating the inferior dog ear seen in a vertical-only incision. The Paisley technique simply uses the concept of a superior medial pedicle to help with bottoming out and a lateral incision to eliminate dog ear or revision rates experienced with a vertical-only incision technique, while allowing high-volume resections and eliminating the medial incision. The study supports the Paisley technique as a safe alternative with the potential benefit of eliminating the medial scar seen using the Wise pattern.^1^ I do find the vertical incision-only breast reduction technique falls short because of potentially higher revision rates of the inferior dog ear deformity. The Wise pattern eliminates this problem, while still allowing the use of superomedial pedicle with its advantages. The Paisley technique eliminates the dog ear deformity of the vertical incision and attempts to minimize the horizontal scar of the Wise pattern, while mostly using a superomedial pedicle with its associated advantage. This may well be a safe and aesthetically pleasing alternative technique in breast reduction surgery.
